# Fuchs endothelial corneal dystrophy: current perspectives on diagnostic pathology and genetics—Bowman Club Lecture

**DOI:** 10.1136/bmjophth-2022-001103

**Published:** 2022-07-28

**Authors:** Caroline Thaung, Alice E Davidson

**Affiliations:** 1 Moorfields Eye Hospital, London, UK; 2 Department of Eye Pathology, University College London Institute of Ophthalmology, London, UK; 3 University College London Institute of Ophthalmology, London, UK

**Keywords:** Cornea, Dystrophy, Genetics, Pathology

## Abstract

Fuchs endothelial corneal dystrophy (FECD) was first described over a century ago. Since then, we have learnt much about its clinical manifestations, surgical and non-surgical treatment, microscopic appearance and pathogenesis. Over the past decade, significant advances have been made with respect to our understanding of FECD genetics. This progress now enables us to appreciate that FECD in fact describes multiple entities with distinct underlying genetic causes. For example, an early-onset and rare form of the disease has been attributed to missense mutations in the *COL8A2* gene, whereas the vast majority of late-onset cases can be attributed to a non-coding repeat expansion within the *TCF4* gene.

FECD is one of the most common indications for corneal transplantation. In recent years, attention has turned to alternative treatment techniques that do not depend on donor tissue supply. The design and development of these non-surgical treatment approaches have benefited from increased knowledge of pathogenesis.

This review will cover our current knowledge about the histology and genetics of FECD, and how combining these interdisciplinary approaches might may improve diagnostic accuracy and aid the development of therapeutics for this common and visually disabling disease.

## Introduction

Fuchs endothelial corneal dystrophy (FECD) is one of the most common indications for corneal transplantation worldwide.^1^ Current treatment is by corneal grafting, whether full-thickness (penetrating) keratoplasty, or by transplanting posterior tissue only. In the UK at least, the recommendation is to submit removed corneal material for histological examination.^2^

Clinical examination and in vivo imaging and laboratory examination of removed corneas are well-established methods of evaluating cases of suspected FECD. Genetic screening is not yet routinely applied in a standard clinical care setting. However, this is anticipated to change as our understanding of the distinct genetic causes of disease and their impact on prognosis and treatment options evolve.

This review will cover what we know about the histology of FECD and its genetic causes, and how this knowledge might be applied in future to allow opportunities for prognostication and treatment.

## What do we mean by FECD?

The term is used to refer to at least two clinical entities, which have common features of endothelial cell dysfunction and Descemet membrane abnormalities. They manifest clinically as worsening vision and corneal oedema and with a slit-lamp appearance of cornea guttata. With the benefit of genetic characterisation, we know that late-onset (or adult-onset) FECD and the much rarer early-onset FECD are distinct diseases, with differing underlying molecular mechanisms. Current evidence shows that early-onset FECD is attributed to missense mutations in the extracellular matrix encoding gene *COL8A2*,^3^ while late-onset FECD is genetically heterogeneous.^4^ However, a significant majority of the late-onset cases have a shared underlying genetic cause of disease: expansion of a triplet repeat element (termed CTG18.1) situated within an intronic region of the transcription factor encoding gene *TCF4*.^4 5^ There are still unexplored gaps in our knowledge and potentially overlaps (both clinically and genetically) between early-onset and late-onset FECD.

## Laboratory specimens for FECD

The Department of Eye Pathology (DEP) is a stand-alone laboratory within the UCL Institute of Ophthalmology, London. It exclusively deals with cellular pathology (histology and cytology) of the eye and surrounding tissues. Corneal specimens include full and partial thickness keratoplasties, small biopsies (eg, infectious keratitis) and impression cytology (in the context of limbal stem cell failure).

Table 1 summarises relevant DEP case numbers over a 24-year period. There is a minor fluctuation between years, but no obvious trend in numbers. Overall, approximately 15% of cases submitted to the DEP are corneas. Of these, approximately 10% are given a diagnosis of FECD. This is in keeping with previous work^6^ and a slightly lower proportion than reported by Ting *et al*.^7^ A caveat when comparing different institutions is that specimens received for examination may not accurately reflect the number of surgeries performed since ophthalmologists may discard tissue or alternatively submit it for research studies rather than diagnostic pathology. The DEP has a large catchment area (Moorfields Eye Hospital in London and many other hospitals within the southern part of England), which varies from year to year. Hospitals with paediatric corneal services may handle their cases in-house, which is a factor when considering early-onset FECD.

**Table 1:** Department of Eye Pathology case numbers over time, showing proportion of corneal specimens and FECD diagnoses

Calendar year	Total cases	Corneas	Corneas as % of total cases	FECD cases	FECD as % of corneas
1998	2442	337	14	41	12
1999	2584	315	12	31	10
2000	2824	508	18	52	10
2001	2877	494	17	59	12
2002	2426	419	17	53	13
2003	2579	445	17	78	18
2004	2822	500	18	85	17
2005	2630	511	19	67	13
2006	2536	497	20	43	9
2007	2547	416	16	41	10
2008	2784	411	15	57	14
2009	2812	466	17	39	8
2010	3017	558	18	67	12
2011	2871	444	15	26	6
2012	3123	596	19	40	7
2013	3115	584	19	50	9
2014	3134	561	18	57	10
2015	3193	491	15	41	8
2016	3206	486	15	35	7
2017	3610	513	14	39	8
2018	3864	529	14	45	9
2019	3953	524	13	54	10
2020	2507	351	14	42	12
2021	3605	492	14	43	9

FECD, Fuchs endothelial corneal dystrophy.

## Descemet membrane and the endothelium

FECD manifests predominantly within Descemet membrane and the endothelium. Descemet membrane is a basement membrane produced by corneal endothelial cells (CECs). It consists of an anterior banded layer (ABL) and a posterior non-banded layer (PNBL). The ABL is laid down prenatally and measures approximately 3 µm thick. On electron microscopy, it has a characteristic latticework of collagen with banding at 110 nm intervals. This appearance is associated with collagen type 8 immunoreactivity,^8^ which supports collagen type 8 as a major component of this layer. The PNBL is laid down by CECs throughout life, and, therefore, thickens with age. By the age of 80 years, the PNBL may be 10 µm thick.^9^

CECs form a monolayer across the posterior surface of Descemet membrane, with their apices on the internal aspect of the cornea (ie, bathed in the aqueous of the anterior chamber). Their major function is to maintain relative corneal stromal dehydration by active fluid and electrolyte transport.

CECs are predominantly a non-proliferative cell population, arrested in the G1 phase of the cell cycle.^10^ Hence, even in the absence of disease, endothelial cell density decreases with age, but this process is accelerated in FECD.^1^ Although a reduction in CECs compromises their active ‘pump’ function, they may still continue to act as a barrier to fluid movement.^11^ Recent advances in single-cell RNA transcriptomics are beginning to shed new light on the diversity of cell types present within this cellular monolayer^12^ and may in future provide insights into the proliferative capacity of certain cell lineages that could be exploited for therapeutic potential.

## Late-onset FECD

In a typical FECD corneal specimen (ie, taken from the central ~9 mm of the cornea) examined with light microscopy, Descemet membrane is thickened with exophytic and/or buried guttae. Endothelial cells are depleted or completely absent.

It is no surprise that few CECs are seen on microscopy since the reason for surgery often relates to compromised endothelial function (ie, corneal oedema). The CECs do not simply reduce in density: they also undergo metaplasia. Their normal cuboidal shape flattens so that they resemble squamous epithelium. This is supported by immunohistochemistry. While normal CECs express CD56 (a marker of neural crest derivation), metaplastic CECs start to express epithelial markers such as CK7 and pancytokeratin while their CD56 immunoreactivity decreases or disappears.^13^

A cell culture study demonstrated that endothelial distribution was related to gutta size, and that CEC cytoplasm did not cover the largest guttae, leaving them ‘bare’ at their apices.^14^ This has previously also been noted in a human case.^11^ One might wonder how CECs can produce guttae that are ‘taller’ than they are. Perhaps CECs occasionally migrate across the apices of guttae, laying down more matrix as they do so. Or perhaps they die after laying down the matrix, and the snapshot of histological examination does not capture this phenomenon. This may be better assessed through in vivo imaging methods such as confocal microscopy.

On light microscopy, the periodic acid-Schiff stain is useful for assessing basement membranes such as Descemet membrane. Thickening and guttae can be seen, and sometimes lamination. However, only a limited level of detail can be appreciated.

Ultrastructural examination of Descemet membrane from FECD specimens demonstrates an additional two layers formed by the abnormal endothelium. The (fetal) ABL is usually normal, suggesting that FECD endothelium has unimpaired function to begin with, but the PNBL may be thinned or lost. A further posterior banded layer (PBL) is present posterior to the PNBL, and then a posterior fibrillar layer (PFL), on which the remaining CECs are distributed.^6 15–18^ Other workers have previously suggested the existence of five layers in FECD, with a border layer lying between the PBL and the PFL.^1 19^

The characteristic guttae of FECD are formed from the material of the PBL. Similarly to the ABL, this layer includes 110 nm-banded collagen, suggesting deposition of collagen type 8. However, it is not as uniform. On electron microscopy, other characteristics have been noted such as spindle-shaped bundles, 10–20 nm diameter fibrils and amorphous substance.^20^ The PBL seems to account for the majority of the Descemet membrane thickening in most cases. Buried guttae are covered by the PFL, which has a smooth or undulating posterior surface on which residual endothelial cells sit.

In late-onset FECD, there is increased deposition of collagens 4 and 8, fibronectin and laminin on the posterior surface of Descemet membrane. In both FECD and pseudophakic bullous keratopathy, a posterior collagenous layer including collagens 4 and 8 and fibronectin is suggested to be produced by CECs which have undergone myofibroblastic transdifferentiation. This posterior-most layer is suggested to reflect a final common pathway before CEC death.^21 22^ Earlier work also comments that the ultrastructural appearance of the PFL is similar to that seen in aphakic bullous keratopathy, so perhaps this layer is less specific and simply indicates general endothelial insult.^15 23^

Although the Hassall-Henle warts of ageing may resemble guttae on light microscopy, they should not cause clinical or histological confusion since they arise at the periphery of Descemet membrane rather than centrally. They are also noted ultrastructurally to have fissures, which are rare in genuine guttae.^19^

Given that FECD is (probably) a primary endothelial disorder, it is highly likely that the more anterior corneal changes are secondary to chronic oedema rather than representing primary pathology. A review of FECD suggests that anterior keratocytes are relatively reduced in density compared with posterior keratocytes, both with in vivo confocal microscopy and on histology.^16^ This author (CT) has not noticed a particular trend for this appearance in FECD corneas although it can be difficult to objectively assess stromal cellularity. Additionally, with increasing use of Descemet membrane stripping surgery rather than penetrating keratoplasty, there is less opportunity to evaluate the stroma in such cases.

**Figure 1:**
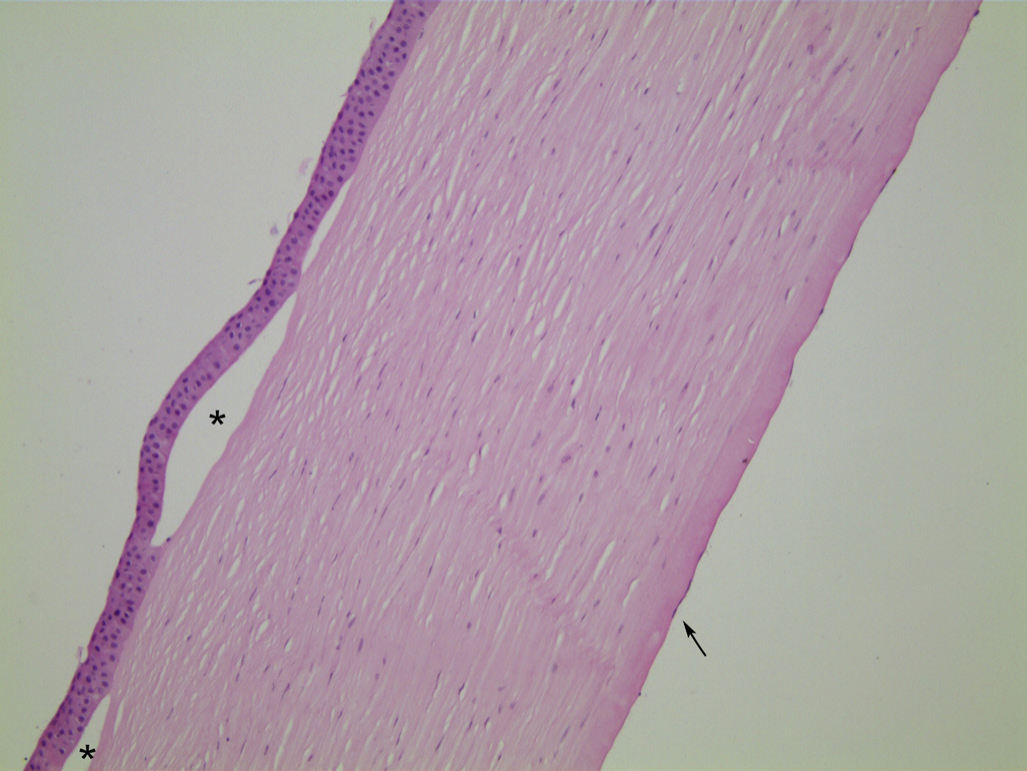
Case 1, H&E stained section, ×10 objective magnification. In this case of histologically classic FECD, the epithelium is lifted off Bowman’s layer to form bullae (asterisks). The stroma is diffusely oedematous, with loss of its normal basketweave texture and reduction in the interlamellar spaces. Endothelial cells are flattened and only visible for short stretches (arrow). FECD, Fuchs endothelial corneal dystrophy.

**Figure 2:**
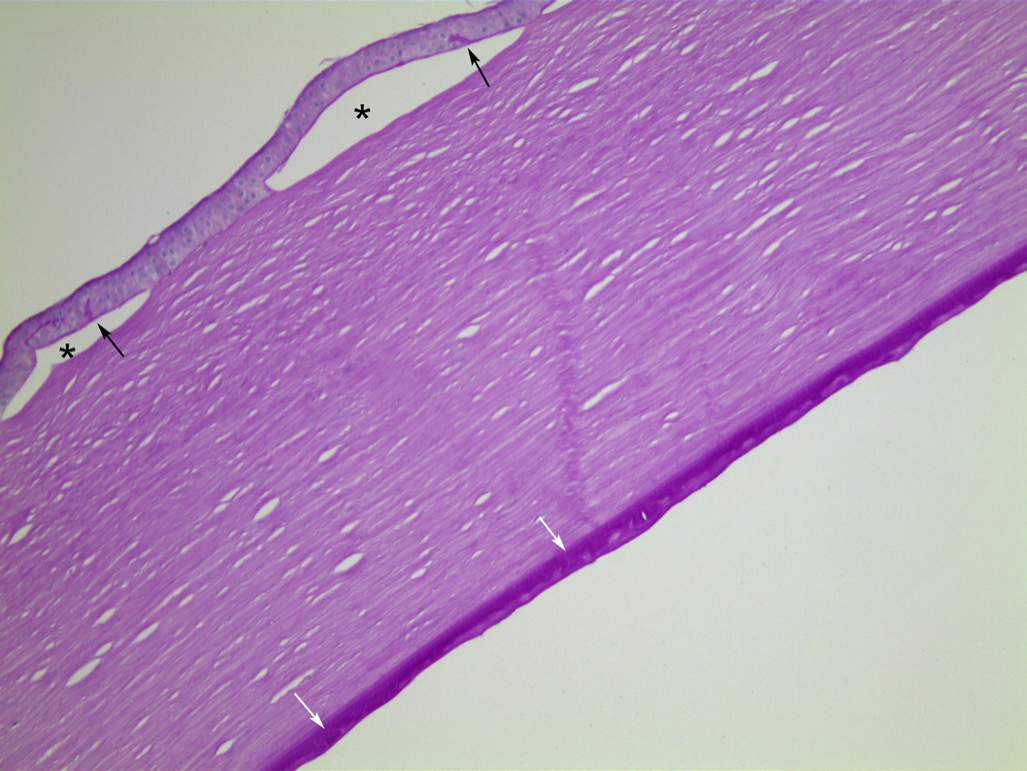
Case 1, PAS stained section, ×10 objective magnification. There are epithelial bullae (asterisks). Additionally, the PAS stain highlights protrusions of basement membrane into the regenerated epithelium (black arrows). Descemet membrane is thickened. Buried guttae (white arrows) are covered by further basement membrane material, giving a fairly regular posterior contour. PAS, periodic acid-Schiff.

**Figure 3:**
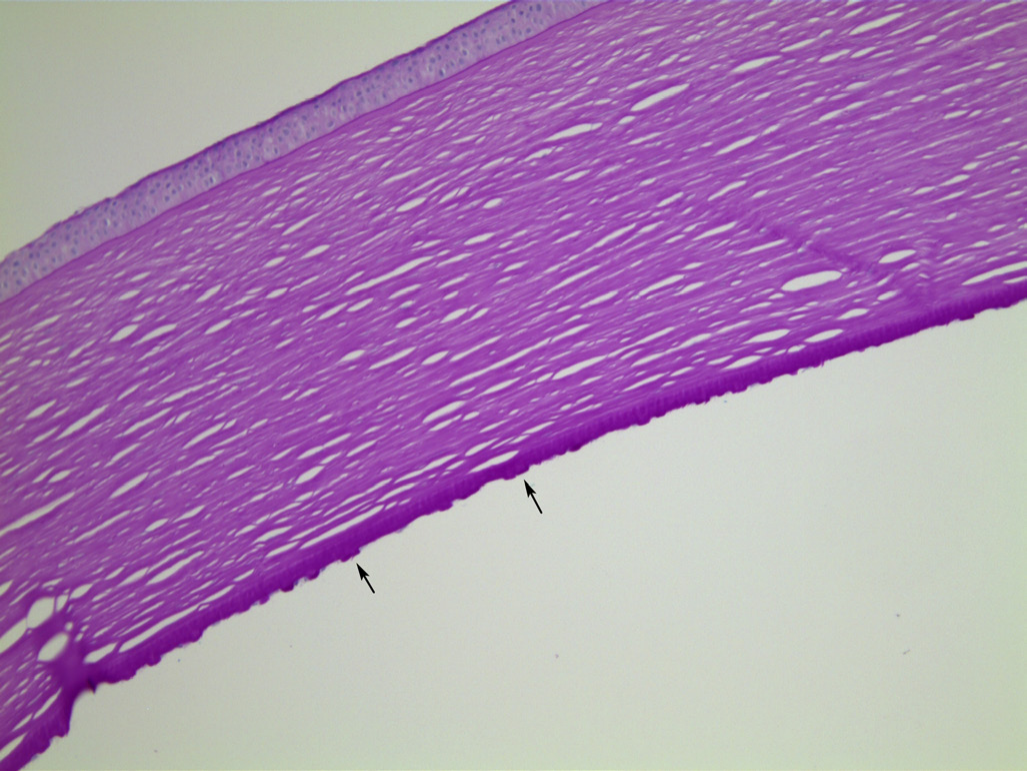
Case 1, PAS stained section, ×10 objective magnification. In this area, Descemet membrane bears large numbers of exophytic guttae (arrows). PAS, periodic acid-Schiff.

Bowman’s layer is morphologically normal in FECD corneas. The epithelium typically shows histological features of chronic oedema. Basal epithelial cells may be pale and swollen. There may be blebbing, as the most superficial cells lift off from the underlying layers, or the entire epithelium may lift to form bullae. With a greater than normal turnover of epithelial cells, there may be thickening of the epithelial basement membrane. Additionally, basement membrane may be laid down within the epithelium (histologically resembling map-dot-fingerprint dystrophy) as well as on Bowman’s layer.^16^ Figures 1–3 demonstrate these classic histological features of FECD.

**Figure 4:**
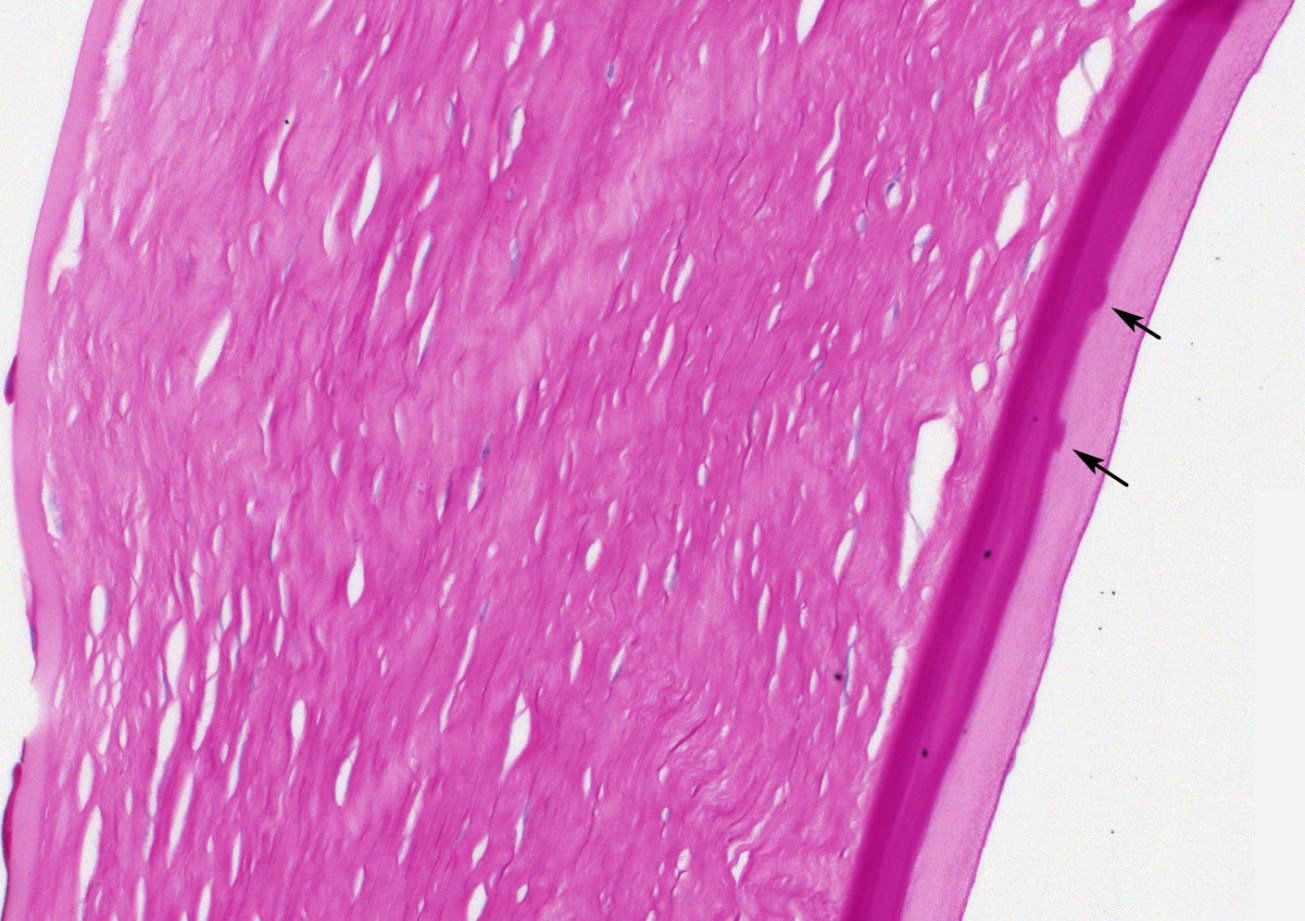
Case 2, PAS stained section, ×20 objective magnification. This is a clinically (not genetically) diagnosed case of FECD, which is histologically non-typical. There is marked thickening of Descemet membrane. Rather shallow, infrequent guttae (arrows) lie on top of a laminated zone. They are covered by a further thick layer with a distinctly different texture. FECD, Fuchs endothelial corneal dystrophy; PAS, periodic acid-Schiff.

The above descriptions apply to histologically ‘typical’ cases of late-onset FECD, where the clinical presentation and presence of guttae (exophytic or buried) both support a diagnosis of FECD. When there is a clinical suspicion of FECD, but guttae are not present (clinically or histologically), interpretation is considerably more challenging. Lamination of Descemet membrane has previously been noted in cases without histological guttae.^24^ However, at that time, knowledge of the underlying genetics was not available. The lamination could potentially have reflected a different disease and/or subtype with a distinct genetic cause. Now that we have (incomplete) knowledge of the underlying genetics of late-onset FECD, we could study the histology of genetically confirmed cases with more confidence about the diagnosis. Figure 4 is an example of a clinically suspected (but not genetically investigated) case of FECD with a non-classic histological appearance.

We can imagine that early on in FECD, diseased endothelial cells lay down a fetal-like PBL, but in an aberrant way, following which they lay down the PFL as a more general preterminal process. But why does this happen? What genetic and subcellular processes drive this abnormal behaviour?

Although FECD is now known to have a strong genetic component, this was not initially recognised. Inherited cases were thought to be a small proportion of total cases.^25^ Even after the turn of the millennium, relatively few patients undergoing surgery for FECD were noted to have a positive family history.^26^ This could be because of the relatively late age of onset (with patients’ parents maybe being deceased), variable severity and other confounding factors such as cataract surgery. Additionally, as FECD is not a life-threatening inherited disease, and treatment is based on symptomatic presentation, perhaps there is less impetus to enquire about family history, especially if such an enquiry is perceived as being ethically complicated due to the need for family counselling.

In 2010, a comparatively small-scale genome wide association study provided compelling evidence for FECD being surprisingly genetically homogenous. The study identified a common polymorphism, located within an intronic region of the *TCF4* gene on Chromosome 18, that conferred up to a 30-fold increased risk for FECD.^27 28^ Two years later, the functional variant underlying this signal of association was identified: expansion of a non-coding intronic triplet repeat element (termed CTG18.1) within *TCF4*.^5^ CTG18.1 repeat length is now well established to be expanded in the majority of FECD cases.^4 5^ Approximately 75% of FECD cases will have at least one expanded allele, defined as ≥50 copies of the repeat. In contrast, unaffected individuals will typically have between 11 and 30 copies of the repeat sequence on both inherited copies of the allele. Incidence of CTG18.1 expansions does, however, notably vary between FECD cohorts investigated to date, depending on ethnicity, with the highest levels of CTG18.1 being recorded within FECD patient cohorts of northern European ancestry.^4^ Mutations in a handful of other genes (including *ZEB1*, *SLC4A11*, *AGBL1* and *LOXHD1*) have been reported to underlie a small number of late-onset FECD cases. However, thus far, the vast majority of late-onset FECD cases negative for CTG18.1 expansions (approximately 20% of cases recruited in an ongoing study at Moorfields Eye Hospital) remain genetically unsolved. Future genomic research efforts are required to shed light on the genetic causes underlying disease in this group.

Given the majority of FECD cases have CTG18.1 expansions, researchers are now focused on determining how expansion of this non-coding element causes FECD. This knowledge will aid the effective design and development of therapeutic interventions. Currently, at least four non-mutually exclusive mechanisms have been proposed. These include (1) dysregulated expression of a subset of *TCF4* transcripts, (2) a toxic accumulation of RNA transcripts transcribed from CTG18.1 expanded alleles and/or (3) non-ATG-dependent translation of repetitive RNA transcripts into toxic repeat peptides and/or (4) age and tissue-specific mechanisms of DNA repeat instability within CECs as covered in a recent review.^4^

## What value lies in providing patients with FECD with a molecular diagnosis?

Because CTG18.1 expansions are so commonly associated with FECD, and harbouring a single CTG18.1 expansion confers a >76-fold risk of developing adult-onset FECD,^29^ there is a strong argument for screening CTG18.1 as a confirmatory step in the diagnostic process. Furthermore, with the knowledge that an index FECD patient has the mutation, family members could be informed about their own risks. And with non-surgical treatments being developed, there may be future opportunities to treat early or even presymptomatic FECD and reduce the need for keratoplasty.

Additionally, CTG18.1-associated FECD falls into the broad category of trinucleotide repeat (TNR) diseases, although it is not a typical example. TNR diseases are largely neurodegenerative, clinically severe (life-shortening) and display autosomal dominant inheritance and anticipation.^30^ Despite the obvious differences, parallels exist between molecular mechanisms underlying FECD and other TNR diseases (eg, RNA toxicity and somatic instability of expanded DNA elements). Given the accessibility of the cornea and the tissue-specific nature of FECD, it is hoped that advances may serve as a model for the rarer, more severe TNR diseases. Conversely, if treatments are developed for other TNR diseases, they could potentially be applied to CTG18.1 expansion-mediated forms of FECD.

## Early-onset FECD

As previously mentioned, the disease currently termed early-onset FECD is a different entity from the far more common late-onset FECD. It typically relates to patients who have been confirmed to harbour missense mutations in the alpha 2 chain of type 8 collagen encoding gene *COL8A2,* which comprises a major component of Descemet membrane. However, genetically unexplained cases have been reported suggesting further genetic heterogeneity may also exist.^3 17 31–34^ Although the term ‘early-onset’ might suggest that this is a disease of childhood, cases may present in adulthood, although at a younger age than typical late-onset FECD cases. On this basis, it is more logical to categorise this disease subtype on the basis of its genetic causes, instead of the age at which the condition is diagnosed.

In common with its late-onset counterpart, early-onset FECD shows guttae on clinical examination (presumably the reason it was termed FECD). However, these guttae have a finer and more regular distribution than noted in late-onset FECD, and they do not coalesce over time.^31^

Electron microscopy shows the ABL to be thickened up to 10 µm (compared with 3–4 µm in normal corneas and late-onset FECD). This thickening supports the initiation of disease in fetal life rather than postnatally. The PNBL is fairly normal. But posteriorly, there is an internal collagenous layer, behind which there is a thick posterior striated layer with horizontally striated material (in contrast to the vertical striations of the ABL).^18^ Guttae are not typically seen with light or electron microscopy.^17^

Additionally, there are wrinkle-like fibrous refractile bodies within the basement membrane matrix, which seem to collocate strongly with COL8A2 and less strongly with COL8A1. Since guttae are not seen on microscopy, it is suggested that the clinical impression is an optical phenomenon because of these refractile bodies. Interestingly, the same study found similar refractile structures in a late-onset FECD case, which had guttae clinically and was not associated with a *COL8A2* mutation.^17^

A slightly later study of three related patients with early-onset FECD and the same *COL8A2* mutation suggests that the thickness of Descemet membrane and the clinical severity of the disease (loss of endothelium and resultant oedema) are correlated. The authors postulate that massively thickened Descemet membrane impairs endothelial nourishment from the stroma, hastening cell loss. Interestingly, two of the three corneas in this study had guttae, with the intermediate severity case lacking them.^35^ It seems unlikely, although not impossible, that guttae regress over time,^15^ and so this variance is difficult to explain.

In early-onset FECD, both COL8A2 and COL8A1 are present in Descemet membrane. However, they are present in a mosaic or clumped pattern rather than the even distribution seen in normal corneas.^17^ There is also an increase in collagen 4, laminin and fibronectin posteriorly in Descemet membrane. It is not clear what contribution the *COL8A2* mutation makes to such deposits, or whether they are simply a non-specific consequence of endothelial disease.^22^ However, it seems plausible that disease-associated *COL8A2* missense mutations could induce conformational shifts in COL8A2 structure that underlie the abnormal distribution of both COL8A2 and COL8A1 in Descemet membrane, given that they form a heterodimer.

## Future prospects

Knowledge about FECD has evolved from clinical appearance through histology, biochemical analysis and genetics studies. We now know considerably more about how and why both early-FECD and late-onset FECD occur. This information is being built on, both for diagnosis and for potential therapeutic utility.

Genetic diagnostic methods are becoming more widely applied, both for inherited diseases (such as neurofibromatosis type 1) and for tumours (eg, BRAF testing in cutaneous and conjunctival melanoma). A list of available tests is kept up to date at: https://www.england.nhs.uk/publication/national-genomic-test-directories/. CTG18.1 screening has not yet been integrated into a diagnostic testing panel. However, given it is now recognised to be by far the most common form of inherited corneal disease, we anticipate that this being added into a future updated version of the diagnostic testing panel in the near future. The currently available version only includes genes that underlie much rarer genetic causes of inherited corneal disease (eg, *COL8A2*), limiting its clinical utility.

In future, it may be possible to treat FECD non-surgically. An array of promising therapeutic approaches is currently being developed to overcome donor tissue reliance. Some have broad application to FECD, irrespective of genetic cause. These include descemetorhexis with/without the addition of the rho-associated kinase inhibitors.^36^ Significant recent advances also include the development of methods to cultivate in vitro transplant-grade human CECs to overcome reliance on human donor tissues required for transplantations.^37^ Promising gene-directed therapies are also on the horizon and offer the potential to treat FECD presymptomatically. These include gene editing approaches that have the potential to directly target causal DNA variants as well as antisense oligonucleotide-based approaches that target features of RNA toxicity attributed to the CTG18.1 expansion^29^ and other more generalised features of TNR-associated disease.^38^

There are still further aspects to explore. For example, do all late-onset FECD cases with the CTG18.1 mutation have guttae on clinical examination and histology? Conversely, is ‘non-guttae’ FECD truly a distinct entity? As further genetic causes of FECD are elucidated, this question could be applied more widely. Since early-onset and late-onset FECD are so dissimilar, it may be time to re-examine nomenclature and consider a gene-centric system. And perhaps even late-onset FECD will eventually be shown to consist of different entities with different pathogenesis, relevance, outlook and treatment.
